# Toxins Utilize the Endoplasmic Reticulum-Associated Protein Degradation Pathway in Their Intoxication Process

**DOI:** 10.3390/ijms20061307

**Published:** 2019-03-15

**Authors:** Jowita Nowakowska-Gołacka, Hanna Sominka, Natalia Sowa-Rogozińska, Monika Słomińska-Wojewódzka

**Affiliations:** Department of Medical Biology and Genetics, Faculty of Biology, University of Gdańsk, Wita Stwosza 59, 80-308 Gdańsk, Poland; jowita.nowakowska@phdstud.ug.edu.pl (J.N.-G.); hanna.sominka@phdstud.ug.edu.pl (H.S.); natalia.sowa@phdstud.ug.edu.pl (N.S.-R.)

**Keywords:** AB-toxins, endoplasmic reticulum (ER), ER-associated degradation (ERAD)

## Abstract

Several bacterial and plant AB-toxins are delivered by retrograde vesicular transport to the endoplasmic reticulum (ER), where the enzymatically active A subunit is disassembled from the holotoxin and transported to the cytosol. In this process, toxins subvert the ER-associated degradation (ERAD) pathway. ERAD is an important part of cellular regulatory mechanism that targets misfolded proteins to the ER channels, prior to their retrotranslocation to the cytosol, ubiquitination and subsequent degradation by a protein-degrading complex, the proteasome. In this article, we present an overview of current understanding of the ERAD-dependent transport of AB-toxins to the cytosol. We describe important components of ERAD and discuss their significance for toxin transport. Toxin recognition and disassembly in the ER, transport through ER translocons and finally cytosolic events that instead of overall proteasomal degradation provide proper folding and cytotoxic activity of AB-toxins are discussed as well. We also comment on recent reports presenting medical applications for toxin transport through the ER channels.

## 1. Introduction

**Figure 1:**
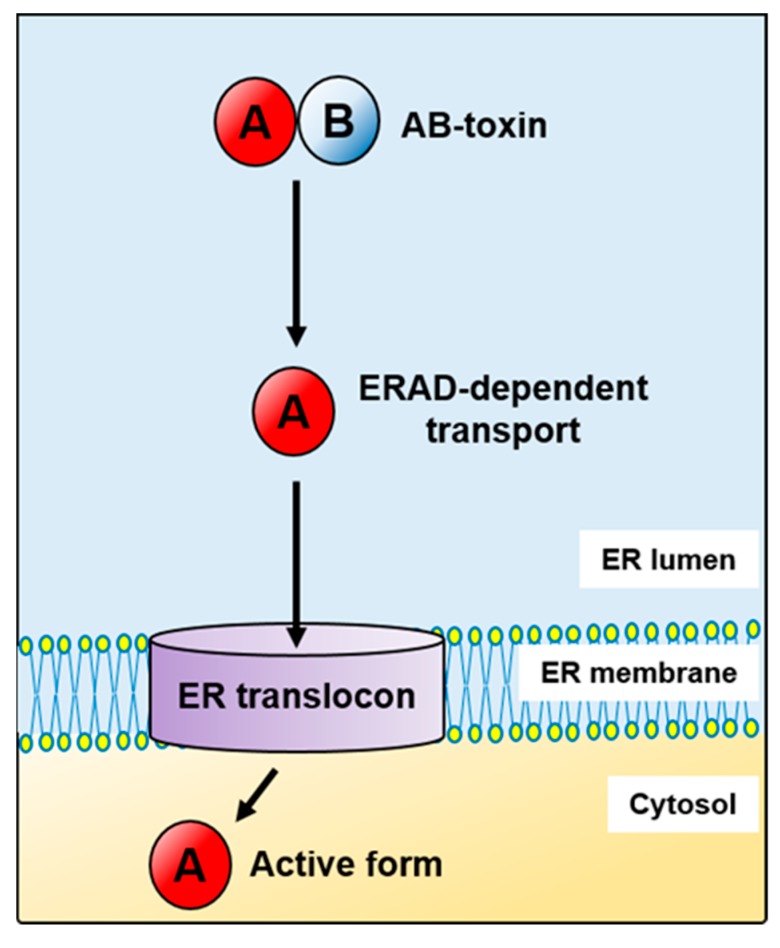
AB-toxins subvert the endoplasmic reticulum-associated protein degradation pathway (ERAD) in their transport from the ER to the cytosol.

Endoplasmic reticulum (ER) is a major cellular protein folding compartment that regulates biosynthesis, assembly and trafficking of most secretory and membrane proteins [1]. In order to maintain the fidelity of essential cellular functions, the ER is dedicated to a stringent quality control system (ERQC) which enables folding and modification of proteins and eliminates terminally misfolded polypeptides through ER-associated degradation (ERAD) or autophagy [2]. ERAD can be divided in four primary phases: (1) recognition (substrate recognition within the ER and direction to the retrotranslocon), (2) retrotranslocation (substrate transport across the ER membrane), (3) membrane unload (release of the substrate from the ER membrane into the cytosol) and (4) degradation (ubiquitin–proteasome dependent degradation) [2,3,4]. During all of these steps ER molecular chaperones and associated factors, both luminal and membrane-bound, ER translocons, as well as diverse cytosolic factors, are crucial for substrate driving through ERAD. However, disposal of different types of substrates, for example, soluble, membrane-bound, glycosylated or non-glycosylated, can be regulated by distinct ERAD pathways that differ in the set of factors involved in ERAD [5,6,7]. Moreover, work on *Saccharomyces cerevisiae* had shown that misfolded ER proteins are degraded by three different ERAD pathways (ERAD-L, -M and -C), depending on whether their misfolded domain is localized in the ER lumen, within the membrane or on the cytosolic side of the membrane [8,9,10]. There is also evidence that ERAD controls degradation of certain folded proteins, including MHC I and CD4. In these cases adaptor-mediated substrate recognition is employed, as MHC I molecules are bound by US11 protein encoded by the human cytomegalovirus, whereas CD4 are targeted for degradation in cells expressing the HIV-encoded ER membrane protein Vpu [9,11,12,13,14,15]. The ERAD strategy of using substrate-specific adaptors is not controlled exclusively by viral encoded proteins. Rhomboids are classified as serine proteases, conserved across all kingdoms of life. A subgroup of rhomboid-like proteins that lack essential catalytic residues, “iRhoms” [16], can target epidermal growth factor receptor (EGFR) for proteasomal removal by ERAD in *Drosophila* [17]. A substrate specific adaptor also functions in the ERAD regulation of HMG-CoA reductase (HMGCR), a key enzyme of the sterol biosynthetic pathway [18]. It has been also reported that regulated degradation of IRE1α and ATF6, important sensors of the unfolded protein response (UPR), is controlled by ERAD [19,20,21]. All of these observations highlight the role of ER-associated degradation in cellular homeostasis and indicate that this process may control the complexity of ER-related functions. Despite complicated and diverse ERAD mechanisms and pathways, cellular significance of this process should be considered in a much broader spectrum. A group of AB-toxins have evolved mechanisms to exploit ERAD for their own benefit (Figure 1). These toxins have an overall similar structure, which typically consists of a single enzymatically active A subunit (chain) and a single or multiple membrane binding B subunit recognizing particular cell surface glycolipids, glycoproteins or receptor proteins. After cell binding and endocytosis, toxins are trafficked in a retrograde manner through the Golgi apparatus and into the ER before reaching the cytosol or the host cell nucleus.

**Figure 2:**
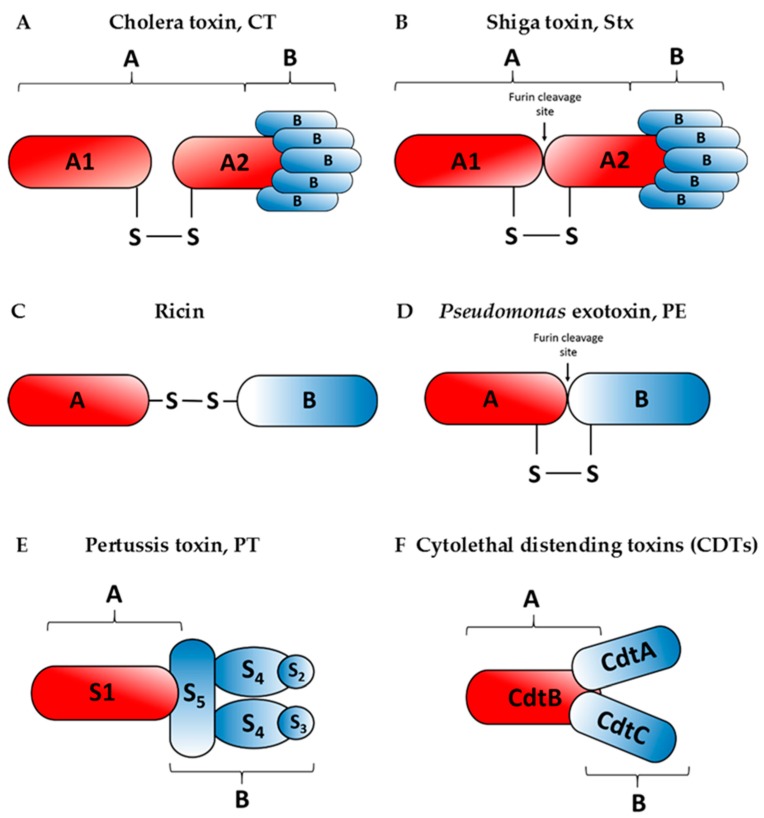
Schematic structures of the cholera toxin, CT (**A**), Shiga toxin, Stx (**B**), ricin (**C**), *Pseudomonas* exotoxin, PE (**D**), the pertussis toxin, PT (**E**) and cytolethal distending toxins, CDTs (**F**). Enzymatically active moieties are indicated as A, whereas binding moieties are indicated as B. Names of particular subunits of the A and B moieties for each toxin are marked inside the subunit structures. In case of the cholera toxin, the A subunit is cleaved before reaching the target cells which is indicated on the diagram by separation of the A subunits.

The first report suggesting a link between the cell cytosol entry of toxins and the ERAD pathway came in 1997 [22]. Then, Rapak and co-workers established an excellent assay for analysing transport of ricin, a plant toxin, from the ER to the cytosol [22]. However, at that time they were not able to address the question as to how the toxin enters the pathway or how it escapes degradation. The fact that toxins avoid effective ubiquitination and thus they are transported to the cytosol without being directed for proteasomal degradation makes them untypical ERAD substrates. Over the past two decades, our knowledge on ERAD-dependent toxin transport to the cytosol has significantly expanded. The first three ERAD steps: recognition, retrotranslocation and membrane unload are generally common for misfolded proteins, endogenous ER substrates and toxins. However, in the case of toxins, they fold properly after transport to the cytosol in order to express their cytotoxic activity (Figure 1). Toxins that hijack the host cell ERAD pathway for their transport from the ER to the cytosol include: the cholera toxin (CT) (Figure 2A), *E. coli* heat-labile enterotoxin (LT), Shiga and Shiga-like toxins (Stx, SLTs) (Figure 2B), ricin (Figure 2C), *Pseudomonas* exotoxin (PE) (Figure 2D), the pertussis toxin (PT) (Figure 2E) and cytolethal distending toxins (CDTs) (Figure 2F) (for review see for example, [23,24,25,26]). Despite similar A-B subunit composition of these toxins, they differ in their structural arrangement and the mode of action. 

The cholera toxin produced by *Vibrio cholerae* is responsible for causing massive, watery diarrhoea characteristic of a cholera infection [27]. Structurally, the CT holotoxin consists of a homopentameric, cell-binding B subunit (CTB) that is noncovalently associated with a single catalytic A subunit (CTA). After proteolytic cleavage, which likely occurs at the target cell surface [28], CTA is separated into two domains: the enzymatic CTA1 and CTA2, linked by a disulphide bond and noncovalent interactions (Figure 2A). CTA2 forms an extended alpha helix which is inserted into the central pore of the B subunit ring. Catalytically active CTA1 of cholera toxin induces ADP-ribosylation of the Gs alpha subunit (Gα*_s_*) proteins using NAD. 

The *Escherichia coli* heat-labile enterotoxin is closely related to the cholera toxin. Its two subunits are structurally and functionally similar, sharing approximately 80% homology with CT [29]. Similarly to the cholera toxin, the A subunit of LT ADP-ribosylates the α subunit of the Gs GTP-binding proteins, resulting in constitutive activation of adenylate cyclase and production of 3′,5′-cyclic AMP (cAMP) [30]. In addition to its effects on chloride secretion, heat-labile enterotoxin also binds other substrates: lipopolysaccharide on the surface of *E. coli* cells and A-type blood antigens [31]. Two antigenically different forms of LT are known: LT-I and LT-II. The prototypical class of LT is considered to be LT-I. LT-I and LT-II have similar structure, however the B subunit of LT-II shares little sequence similarity to LT-I. It has been reported that strains expressing LT-II are not often isolated from human patients [32].

The Shiga toxin is produced by *Shigella dysenteriae*, whereas Shiga-like toxins are secreted by certain strains of *Escherichia coli* (STEC: Shiga-like toxin producing *Escherichia coli*). It has been reported that Shiga-like toxins can be also produced by *Citrobacter freundii*, *Aeromonas hydrophila*, *Aeromononas caviae* and *Enterobacter cloacae* [33]. Similarly to the cholera toxin, these toxins have AB_5_ subunit configuration (Figure 2B). Five identical subunits of the binding moiety (B) recognize specific glycolipids on the host cell, specifically the globotriaosylceramide (Gb3). The enzymatic A subunit, which is non-covalently connected to the binding moiety, is cleaved asymmetrically into an A1 and A2 peptides held together by a disulphide bridge. This cleavage is processed by furin at a low pH, suggesting that it occurs early in the transport pathway. Within the ER, the Shiga toxin A1 fragment dissociates from the A2 fragment and the B subunits [34,35]. The A1 subunit is a potent protein synthesis inhibitor which acts by cleaving a single adenine residue in 28S rRNA of the 60S eukaryotic ribosomal subunit [34]. 

The pertussis toxin is an AB_5_-type exotoxin produced by *Bordetella pertussis*, the etiological agent of whooping cough. The B oligomer ring-shaped structure is composed of S2, S3, two S4 and an S5 subunits (Figure 2E), which bind to various (but mostly unidentified) glycoconjugated molecules at the surface of target cells [36,37]. S5 serves to link the two dimers, S2–S4 and S3–S4. The enzymatic activity of PT resides in the A subunit, also known as S1. Once in the cell cytosol, S1 hydrolyses cellular NAD and catalyses ADP-ribosylation of GTP-binding regulatory proteins involved in signal transduction in the eukaryotic cells [37].

Both, the *Pseudomonas* exotoxin A (Figure 2D) and ricin (Figure 2C) belong to the two-component AB-toxin family, composed of an A domain with enzymatic activity and a B domain serving as the cell binding subunit [38,39]. *Pseudomonas* exotoxin A is the most toxic virulence factor of the pathogenic bacterium *Pseudomonas aeruginosa*. Ricin is an extremely potent protein toxin isolated from castor beans, the seeds of the castor plant, *Ricinus communis*. The A subunits of both toxins inhibit protein synthesis after their transport from the ER to the cytosol. The PE fragment ADP-ribosylates the eukaryotic elongation factor-2 (eEF-2) at the eukaryotic ribosomes [40]. On the other hand, the ricin A chain has an *N*-glycosidase activity, as it specifically removes an adenine residue located in an exposed, strictly conserved loop in 28S rRNA, the so-called sarcin-ricin domain (SRD). This loop is part of the recognition/binding site for the eukaryotic elongation factor 1 (eEF-1) and the eukaryotic elongation factor 2 (eEF-2) complexes [41,42].

Cytolethal distending toxins are bacterial proteins widely distributed among Gram-negative bacteria, including *Escherichia coli*, *Campylobacter* spp., enterohepatic *Helicobacter* spp., *Actinobacillus actinomycetemcomitans* and *Haemophilus ducreyi*. These toxins were proposed to belong to a bacterial effectors termed “cyclomodulins” that interfere with the eukaryotic cell cycle. Expression of these toxins is associated with severity of disease [43]. CDTs function as complexes of three distinct protein subunits, named alphabetically in the order that their coding genes appear in the *cdt* operon (*cdtA*, *cdtB*, *cdtC*) [44]. They are considered as AB_2_ type of toxins, CdtB functions as the enzymatic A subunit, whereas CdtA and CdtC operate together as the cell-binding B moiety of AB-toxins that deliver CdtB into cells [43,44,45,46] (Figure 2F). CdtB possesses DNase I-like activity responsible for inducing DNA damage within the nuclei of intoxicated cells [47]. This damage triggers a signalling pathway that involves different protein kinases which results in a block before cells enter mitosis.

All of the described toxins have different intracellular targets and thus exert very diverse effects on eukaryotic cells. They induce overall cytotoxicity, cell apoptosis and tissue damage or generate more subtle alternations in the host through changes in the cell cycle progression. Despite all of these differences, their cytotoxicity can be regulated by the ERAD system. In this review, we describe mechanisms that direct toxins through the ERAD pathway. We discuss the role of toxin mutations or instability in their transport from the ER to the cytosol. We also describe how regulated toxin transport across the ER membrane can rescue misfolded mutant proteins from degradation to increase their cellular function.

## 2. Toxin Recognition and Disassembly in the ER

Delivery of the toxins to the ER has been demonstrated by: i) microscopy observations; ii) analysis of the role of KDEL, the ER retention signal; iii) indication that genetically modified toxins are glycosylated by specific enzymes present in the ER; and iv) demonstration that toxins interact with the ER proteins. The first evidence that an endocytosed toxin can be transported not only to the trans-Golgi network but further to the ER came from experiments with the Shiga toxin [48]. Treatment of A431 cells with butyric acid significantly sensitized these cells to this toxin. Under such conditions, the Shiga toxin could be visualized in the ER by electron microscopy. Three years later, it was demonstrated that *E. coli* heat-labile enterotoxin directly interacts with the endogenous KDEL receptors which implied that these toxins may require retrograde movement through the Golgi cisternae and the ER for efficient and maximum biological activity [49]. Next, experimental data indicated that the glutamate residue of KDEL improves the cytotoxicity of *Pseudomonas* exotoxin A by increasing its binding to a sorting receptor which transports the toxin from the Golgi apparatus to the ER [50]. Other evidence that the KDEL retrieval system is exploited not only by PE [51], but also by the cholera toxin [52], was also provided. Moreover, it was shown by electron microscopy that horseradish peroxidase-labelled cholera toxin is transported to the ER in thapsigargin-treated A431 cells [53]. In the case of ricin, a genetically modified toxin containing the C-terminal site for tyrosine sulfation (a trans-Golgi-specific activity) and three partly overlapping N-glycosylation sites (an ER-specific activity), was produced [22]. Such modified A chain became sulphated but also core glycosylated, indicating retrograde transport to the ER. The first indication that the pertussis toxin is transported to the ER was based on the observation that PT delivers major histocompatibility complex (MHC) class I-binding epitopes to MHC class I molecules without utilizing the endogenous cytosolic antigen-processing machinery. Therefore, it was suggested that the pertussis toxin can deliver the epitopes directly to nascent class I molecules in the ER [54]. Moreover, cytotoxic activity of PT was observed in cells transfected with a genetic construct encoding the S1 subunit fused with an ER signal peptide [55]. These results were consistent with a pathway in which the pertussis toxin is trafficked to the ER. Final confirmation that this toxin is transported to the ER comes from experiments in which, similarly to experiments performed with ricin [22], peptide target sites for tyrosine sulfation and N-glycosylation were added [56]. In PT experiments, the S1 (A subunit) and S4 (B subunit) were modified. The obtained results indicated that both, the S1 and S4 subunits of PT are retrogradely transported through the TGN to the ER, suggesting additionally that PT is transported to the ER as an intact holotoxin. Evidence that cytolethal distending toxins are trafficked to the ER was provided by experiments with *Haemophilus ducreyi* CDT (HdCDT) [57]. In these experiments a modified holotoxin was used. This holotoxin contained CdtB subunit that carries either a sulfation site or a sulfation site and three partially overlapping N-linked glycosylation sites at the C-terminus. These studies demonstrated that the active subunit moves via the trans-Golgi and it is retrogradely translocated to the ER. 

Thus, toxins are transported to the ER as fully-folded, stable AB-holotoxins. In order to be directed to the cytosol by ERAD, they have to be disassembled and recognized by different ER molecular chaperones that move them to a particular translocation channel present in the ER membrane. 

### 2.1. Unfolding and Release of A Fragments from the Holotoxins

Specific folding enzymes that operate in the ER include thiol oxidoreductases of the protein disulphide isomerase (PDI) family (for review see for example, [58]). Their basic, critical role in the maturation of the majority of proteins that traffic through the ER is the formation of disulphide bonds (S-S) by oxidation of free sulfhydryl (SH) groups of cysteine residues. However, to obtain native disulphides of ER proteins, the oxidoreductases catalyse their reactions by acting as oxidases and isomerases [59]. This allows proteins to quickly find the correct arrangement of disulphide bonds during the folding process by rearrangement of non-native linkages. Thus, the PDI family members that act to catalyse protein folding are classified as foldases. In the case of toxins, these enzymes are involved in the unfolding of enzymatically active toxin subunits and can catalyse reduction of the internal disulphide bonds connecting the A and B subunits of holotoxins before transport of the A fragment through the ER membrane. 

**Figure 3:**
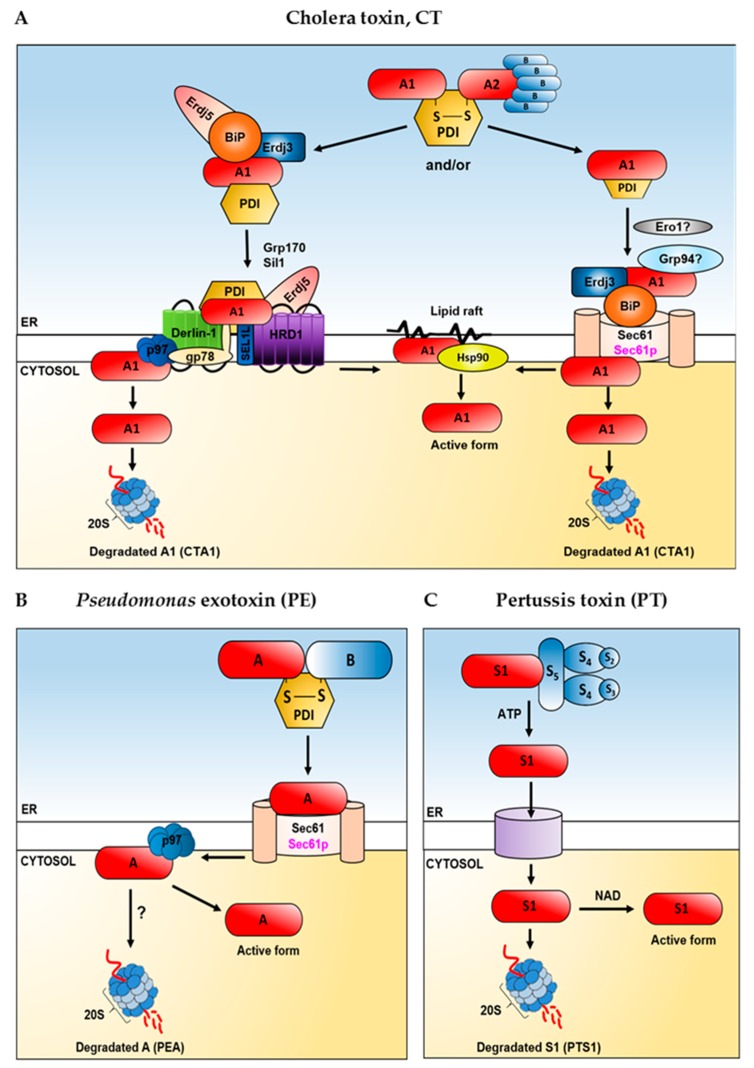
A schematic proposal on how toxins utilize the ER and cytosolic host factors in their transport to the cytosol in the mammalian or yeast cells. Yeast proteins are shown in magenta, mammalian proteins are shown in black. Cholera toxin, CT (**A**), *Pseudomonas* exotoxin, PE (**B**), pertussis toxin, PT (**C**). In case of the cholera toxin (**A**) two alternative mechanisms (indicated as “and/or”) that may operate in its transport out of the ER are considered. Role of Ero1 in release of the toxin from PDI is not clear. There are some contradictory data concerning interactions of Grp94 with CTA1. For detailed description and references, see the main text. There are no data confirming proteasomal degradation of the A subunit (PEA) of *Pseudomonas* exotoxin (**B**). In (**A**–**C**), 20S refers to the core particle of the proteasome. For detailed description of the overall role of ERAD factors in toxin transport to the cytosol and appropriate references, see the main text.

These PDI features are fully exploited by the cholera toxin. Reductive release of the enzymatic A1 chain of a proteolytically nicked CTA has been shown to require the activity of PDI in vitro [60] (Figure 3A). A more detailed study indicated that reduction of the disulphide bridge between A1 and A2 fragments is preceded by unfolding of the A subunit, thus PDI has been proposed to act in vitro as a binding chaperone and as an unfoldase [61].

Interestingly, the A subunit needs to be cleaved into the A1 and A2 fragments for unfolding to occur. Here, the PDI does not act as an oxidoreductase but as a redox-dependent chaperone. It binds the toxin that is in the reduced state, while in the oxidized state the substrate is released [61]. In the presence of reduced PDI, the A1 subunit becomes markedly trypsin sensitive. This feature seems to be specific for PDI, as another member of the PDI family, ERp72, stabilizes the A subunit in a trypsin-resistant form [62]. On the other hand, the cholera toxin release from PDI is mediated by the action of Ero1 (Figure 3A), which oxidizes the COOH-terminal disulphide bond in PDI. The PDI and unfolded toxin complex is first bound at the lumenal side of the ER membrane via PDI and then oxidation of PDI by Ero1 and release of the toxin from PDI is conducted [63]. However, the molecular mechanism of the cholera holotoxin disassembly seems not to be so obvious. The Ken Teter group has published their experiments showing that PDI does not unfold CTA1 [64] but instead unfolds itself upon contact with CTA1 [65]. They postulate that unfolded PDI acts as a wedge to dislocate the already reduced CTA1 from its holotoxin. This process was specific for PDI as two other oxidoreductases (ERp57 and ERp72) remained folded in the presence of CTA1 and did not displace reduced CTA1 from its holotoxin. The oxidoreductase activity of PDI was not necessary for CT disassembly [65]. Moreover, the release of PDI from CTA1 does not require Ero1p (Figure 3A) but instead results from the spontaneous unfolding of CTA1 which occurs after its dissociation from the holotoxin at a physiological temperature [64,66,67]. Recently published experiments had shown that the PDI-induced conversion of CTA1 into a protease-sensitive state [61] is not an enzymatic process and it is not functionally linked to the CT disassembly [68].

Disassembly of the *Pseudomonas* exotoxin A is generally similar to the cholera toxin. Furin-nicked PE is susceptible to unfolding and disulphide bond A subunit reduction. Unfolded PE, which is sensitive to trypsin, is subjected to the PDI-mediated reduction (Figure 3B). However, PDI by itself does not have the ability to unfold this toxin [69]. When subcellular fractions from toxin-sensitive cells were incubated with nicked PE, the toxin unfolding and reducing activities were present in the membrane fraction but not in the soluble fraction. 

In the case of pertussis toxin, ATP is required for displacement of the PTS1 subunit from its noncovalent association with PTB [70] (Figure 3C). This process can take place exclusively in the ER because endoplasmic reticulum is the only endomembrane compartment that contains ATP [71]. This nucleotide can be bound in the central pore of the B-oligomer and makes extensive interactions with the protein [72], changing conformation of PTB. This triggers dissociation of the pertussis toxin subunits. Reduction of the intramolecular PTS1 disulphide bond also alters conformation of the A subunit such that it no longer readily associates with the B oligomer of the toxin [73]. Disassembly of the pertussis holotoxin leads to the spontaneous unfolding of PTS1, which become a thermally unstable protein upon dissociation from PTB [74]. Moreover, inhibition of PTS1 unfolding via chemical chaperones, substantially decreased the cytosolic pool of PTS1 and blocked PT-associated intoxication [75]. It can be concluded that the thermal unfolding of dissociated PTS1 triggers its ERAD-mediated translocation to the cytosol.

**Figure 4:**
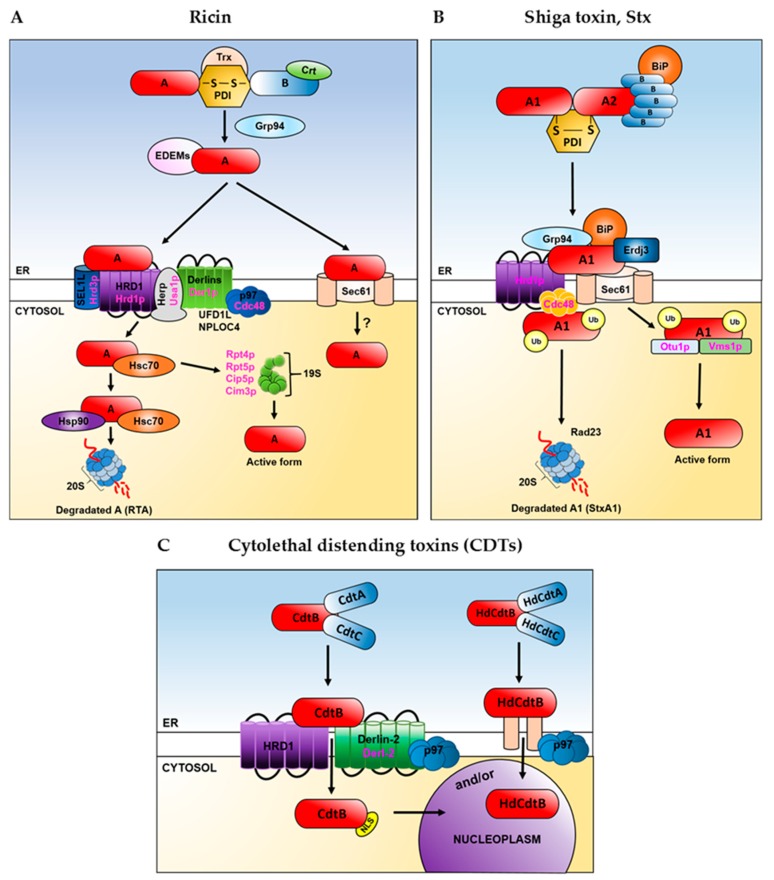
A schematic proposal on how toxins utilize the ER and cytosolic host factors in their transport to the cytosol in the mammalian or yeast cells. Yeast proteins are shown in magenta, mammalian proteins are shown in black. Ricin (**A**), Shiga toxin, Stx (**B**) and cytolethal distending toxins, CDTs (**C**). Role of Sec61 in ricin (**A**) transport to the cytosol is not clear. For detailed description and references, see the main text. Trx–thioredoxin, Crt-calreticulin, EDEMs refers to EDEM1, EDEM2, EDEM3. Derlins refers to Derlin-1, Derlin-2, Derlin-3. 20S refers to the core particle, whereas 19S refers to the regulatory particle of the proteasome. For Shiga toxin (**B**), some information about the role of the ER and cytosolic ERAD proteins comes from experiments performed with the usage of Shiga-like toxin, SLTxA1(see the main text). Ub-ubiquitin. In case of cytolethal distending toxins (**C**) two alternative mechanisms (indicated as “and/or”) that may operate in these toxins’ transport from the ER to the nucleoplasm are considered. Direct transport from the ER to the nucleoplasm can be used by *Haemophilus ducreyi* A chain, HdCdtxB (see the main text). NLS-nuclear localization signal. In (**A**–**C**) for detailed description of the overall role of ERAD factors in toxin transport out of the ER and appropriate references, see the main text.

The reductive separation of the ricin subunits requires a remodelling of the holotoxin structure to open the interface between RTA and RTB and allow reductive cleavage [76]. It has been demonstrated that PDI interacts with the ricin B chain (RTB) and can both, reduce and form the disulphide bond linking the ricin A chain (RTA) and RTB in vitro [77] (Figure 4A). Reduction of the disulphide bond allows enzymatically active RTA to be released from lectin RTB. Under certain experimental conditions, formation of the disulphide bond by PDI enabled to arrange heterodimers between endogenous B chains and A chains derived from the reduced holotoxin [77]. For these reasons, cell lines expressing RTB directed to the ER via a mammalian signal peptide were significantly more resistant to intoxication by ricin holotoxin, when compared to cells without ectopically expressed, ER-localized RTB [77]. However, when PDI is depleted from the cells, reductive release of the ricin A chain from holotoxin can still take place, indicating that other oxidoreductases might be also implicated in this process. It was shown that the thioredoxin reductase (TrxR) can lead to reduction of ricin holotoxin by activating the disulphide reductase activity of PDI [78]. PDI and thioredoxin (Trx) could reduce ricin in vitro (Figure 4A) in the presence of TrxR and NADPH. Moreover, TMX, a transmembrane thioredoxin-related protein member of the PDI family, reduces the ricin holotoxin in vitro [79]. It has been demonstrated that the ricin-dependent cytotoxicity was significantly enhanced in TMX-overexpressing cells. It cannot be excluded that TMX, which is localized in the ER membrane, may play a very important role in the reductive activation of ricin and in the subsequent interaction of RTA with components of the ERAD pathway, thereby facilitating the transmembrane movement of RTA and cell intoxication [79]. This hypothesis is supported by earlier observations which indicated that reduction of the disulphide bond serves to activate the catalytic activity of RTA [80]. Affinity-purified proricin did not catalyse the depurination of 28S ribosomal RNA unless it was reduced, when its slight but significant activity was observed. Here, it should be mentioned that contrary to the results described above, ricin holotoxin covalently coupled by a non-reducible thioether bond remained cytotoxic to mammalian cells, suggesting that both subunits may translocate to the cytosol where proteolysis liberates the catalytic fragment [81]. Some evidence suggests that at least partial RTA unfolding is required for its retrotranslocation [82]. Introduction of a disulphide bond into the ricin A chain decreased such holotoxin’s cytotoxicity. Since the changed holotoxin had identical RTB cell binding and RTA catalytic activities as the wild-type holotoxin, it was suggested that reduction in cytotoxicity caused by the introduced disulphide bond resulted from a constraint in the unfolding of RTA. Thus, these results may indicate that RTA unfolding occurs in the ER and is necessary for the membrane translocation of RTA during its entry into the cytosol [82]. This hypothesis was supported by the observation that demonstrated in vitro instability of a native A chain at pH 7.0. Partially unfolded RTA was described as a protease-sensitive, possessing compact secondary structure with disrupted tertiary structure [83]. Thus, it is possible that RTA spontaneously unfolds in the ER lumen, where it can be recognized in a way similar to misfolded proteins. 

The reductive separation of the holotoxin subunits of ricin and the cholera or pertussis toxins, is relatively well studied in comparison to some other ER-delivered toxins. However, since catalytically active A fragments of the heat-labile enterotoxin, Shiga and Shiga-like toxins, are disulphide linked to the rest of the toxin, then it is assumed that a reductive event in the ER is required prior to the A chain transport to the cytosol. It is believed that in case of these toxins, the PDI also unfolds their A subunits [84] (Figure 4B). Shiga and Shiga-like toxin A1 and A2 chains are disulphide linked when they reach the ER. Tam and Lingwood [85] presented data from experiments in which intracellular transport of a fluorescent dual-labelled A and B subunits of Shiga-like toxin (verotoxin) was analysed using confocal fluorescence microscopy. No full-length holotoxin was detected in the cytosol and most cell-associated A subunit remained non-reduced. 

Thus, it was concluded that reduction of disulphide bonds linking the A1 and A2 fragments of Shiga-like toxin, separation from the B subunits and cytosolic translocation must occur in a rapid succession. It was also concluded that reduction of the proteolytically cleaved A subunit could be rate limiting for translocation, that is, subunit separation and translocation probably occur as a continuous, coordinated process.

Cytolethal distending toxins differ in their structure from typical AB-toxins. They do not possess a disulphide bond linking the catalytic A fragment with the rest of the toxin (Figure 2F). However, based on the generality that most proteins require at least partial unfolding to move from the ER to the cytosol, it could be assumed that protein unfolding may be also applied to CDTs [86]. However, it has been demonstrated that the catalytic A moiety (HdCdtB) of the *Haemophilus ducreyi* CDT (HdCDT) is heat-stable [87]. It has been further indicated by cell-based assays that this subunit does not unfold before exiting the ER and that it may be transported directly from the ER lumen to the nucleoplasm (Figure 4C). This would suggest a novel mode of ER exit for HdCdtB and explain distinct structural properties of its catalytic subunit in comparison to other ER-translocating toxins. On the other hand, recently published results demonstrated that CDTs, including HdCDT, require components of the ERAD pathway in their intoxication process [43] (Figure 4C). 

### 2.2. Toxin Interaction with ER Chaperones 

Besides oxidoreductases that belong to the protein disulphide isomerase family [88], which like PDI can discriminate between substrates based on their degree of folding [89], the ER contains specific molecular chaperones, folding enzymes and quality control factors that promote correct folding of newly synthesized polypeptides and ensure that only properly folded and assembled proteins are transported through the secretory pathway (for review see for example, [90,91]). ER chaperones can be informally divided into classical chaperones and carbohydrate-dependent (lectin) chaperones, specific to the ER. Classical chaperones are grouped into several subfamilies, including Hsps of 40, 60, 70, 90 and 100 kDa in size. Interestingly, the ER lumen does not contain members of the Hsp60 (chaperonins) family [91]. Lectin chaperones recognize both, glycans or the bulky hydrophilic extensions, as well as misfolded regions of aberrant proteins [7,91,92]. In order to fully exploit the ERAD machinery to be directed to the ER membrane translocons, toxins interact and utilize not only the PDI or PDI family members but also various reticular chaperones. 

#### 2.2.1. Toxin Interaction with Classical ER Chaperones 

BiP/GRP78 (immunoglobulin heavy chain-binding protein) belongs the Hsp70 family of chaperone proteins. It has been considered as the master regulator of the ER functions [93]. This chaperone participates not only in protein folding and oligomerization but for example, contributes to calcium homeostasis in the ER, plays an important role in the preparation of terminally misfolded ER proteins for ERAD or is involved in regulation of the signal transduction pathway, the unfolded protein response [91,93,94,95,96]. For the chaperone activity of Hsp70 proteins, ATP binding and hydrolysis are essential. Hsp70-ATP exhibits a low substrate binding affinity, whereas Hsp70 bound to ADP represents substrate high-affinity state. [97]. Hsp70 chaperones have low ATPase activity, thus their ATPase cycle is controlled by co-chaperones of the J-domain protein family, which target Hsp70s to their substrates and by nucleotide exchange factors, which determine the lifetime of the Hsp70-substrate complex. BiP co-chaperones that belong to the Hsp40 family, include ERdj1-8 proteins [98] and nucleotide exchange factors, NEFs or GrpE-like families, such as BAP/Sil1 [99] and GRP170 [100]. Grp94, a member of the Hsp90 family, is the most abundant glycoprotein in the ER [91,101]. TorsinA represents the Hsp100 family [102]. 

Experiments that have been performed during the last several years, clearly indicate that the cholera toxin export from the ER to the cytosol strongly depends on the Hsp70 chaperones [103,104,105,106] (Figure 3A). It was shown that CT’s enzymatically active A subunit transport to the cytosol was restored when export-incompetent microsomes, which initially had been depleted of their luminal proteins, were reconstituted with purified BiP [103]. It was also shown that BiP inhibits aggregation of CTA1, thus it is probable that this chaperone protein can keep CTA1 in a soluble, export-competent state [103]. Moreover, it has been demonstrated that treating cells with CT or CTB quickly up-regulates the level of BiP [104]. These data suggest that CT may promote retrotranslocation of the A chain to the cytoplasm by rapidly up-regulating of ERAD proteins, since not only BiP but the level of other proteins involved in the retrotranslocation process was also regulated by the cholera toxin (see below). It can be predicted that if CTA1 transport to the cytosol depends on BiP, it would also rely on Hsp40 co-chaperones and GrpE-like proteins. Indeed, it was shown that a Hsp40 family protein, ERdj3, directly interacts with CTA1 (Figure 3A) and expression of a dominant negative ERdj3 blocks CTA1 translocation into the cytosol and CT intoxication [105]. Moreover, ERdj5, another ER-localized Hsp40, promotes CTA1 transport out of the ER [106] (Figure 3A). In this case, ERdj5 is part of a larger retrotranslocation machinery that captures the cholera toxin once the toxin is released from BiP and directs it to the ER membrane HRD1 complex (see below) (Figure 3A). In addition, it was demonstrated that the ER-resident nucleotide exchange factors, Grp170 and Sil1, induce CTA release from BiP in order to promote toxin retrotranslocation [107] (Figure 3A). Grp170 not only exerts NEF activity but contains a C-terminal holdase domain that functions to prevent protein aggregation [108]. Interestingly, it was suggested that after NEF-dependent release from BiP, the toxin is transferred to a protein disulphide isomerase which unfolds CTA1 and thus allows the toxin to cross the ER translocon. It cannot be excluded that the cholera toxin–PDI interaction occurs entirely downstream of the toxin release from BiP (Figure 3A). In agreement with this suggestion, it was demonstrated that a fraction of PDI is localized proximally to the ER membrane complex, by virtue of PDI’s interaction with Derlin-1 and HRD1 [109,110] (Figure 3A). On the other hand, BiP is located near the Sec61 ER translocon [111] (Figure 3A), which can be also used by CTA1 for transport to the cytosol [112]. It mainly interacts with unfolded substrates [1,113] or can recognize partly folded substrates [114], whereas PDI interacts with a substrate protein at all stages along its folding pathway, also weakly with folded proteins [89]. BiP has been proposed to help keeping the unfolded CTA1 from aggregation in the ER [103] and ERdj3 can be bound to unfolded but not foldedconformations of the isolated CTA1 subunit [105]. Therefore, it might be that interactions between PDI and CT precede interactions with BiP or that the cholera toxin can interact with BiP and PDI at the same time (Figure 3A). Considering the role of other chaperone proteins, it should be noted that retrotranslocation of CTA1 was decreased by downregulation of torsinA, an AAA+ ATPase located within the lumen of the ER and member of the Hsp100 family [115]. The role of Grp94, a Hsp90 protein is not clear (Figure 3A). It has been demonstrated that neither CTA nor CTB interact with Grp94 [106]. On the other hand, it was shown that Grp94 can be bound to CTA1 in an ATP-dependent process that was blocked by specific inhibitors [116]. 

Interestingly, not only the A fragment of toxins may be involved in interactions with chaperone proteins. Shiga-toxin B fragment (StxB) interacts with BiP [117] (Figure 4B). Through this association, the StxA and StxB contact site could be covered, enabling subunit dissociation. Alternatively, low quantities of StxB might be translocated to the cytosol as was previously suggested [118]. The Shiga toxin catalytic A1 subunit strongly interacts with a BiP co-chaperone, ERdj3 [119] (Figure 4B). Furthermore, overexpression or disruption of ERdj3 function generates cellular resistance to the Shiga toxin [120]. StxA was also co-immunoprecipitated with BiP and Grp94 (Figure 4B), both in the presence and in the absence of a cross-linker. However, these interactions were much weaker than those with ERdj3 [119]. 

In contrast to the cholera and Shiga toxins, ricin transport to the cytosol and its cytotoxicity seems to be negatively regulated by BiP [121]. It has been demonstrated that overexpression of BiP inhibited ricin translocation out of the ER and protected cells against this toxin. On the other hand, shRNA-mediated depletion of BiP enhanced toxin transport to the cytosol, resulting in increased cytotoxicity. Interestingly, both the ricin A chain [121] and B chain [122] interact with BiP. It cannot be excluded that BiP may be a part of a bigger protein complex that forms in the ER and inhibits ricin A chain targeting or transport through the ER translocons. Alternatively, ERAD of ricin can be somehow regulated by KDEL receptors and proteins. BiP contains a C-terminal KDEL sequence which is known to act as an ER retrieval signal. Generally, this target peptide sequence prevents the protein from being secreted from the ER and facilitates its return if it is accidentally exported. Ricin does not contain the KDEL sequence, however, introduction of this signal into the A chain increased its toxicity and resulted in a more efficient glycosylation, indicating enhanced transport from the Golgi complex to the ER [123]. One can assume that high overproduction of BiP could result in escape of ricin-BiP complexes out of the ER. These complexes might be disrupted in the Golgi and ricin without the KDEL sequence would be not efficiently transported back to the ER. The cholera toxin, whose transport to the cytosol is positively regulated by BiP [103], has a KDEL sequence [124]. The Shiga toxin does not possess this sequence [124] but in this case, it was only demonstrated that StxA interacts with BiP [119] and it is unknown how overproduction of BiP influences Shiga toxicity. Ricin toxicity does not depend on BiP, however a drug- induced inactivation of Grp94 protects cells against ricin [116,125]. 

Interactions between individual ER chaperones and subunits of other ERAD-dependent toxins have not yet been established. 

#### 2.2.2. Toxin Interaction with Carbohydrate-Dependent ER Chaperones 

The ER hosts a unique class of carbohydrate-dependent chaperones that can recognize various protein substrates in a glycan-dependent but also glycan-independent manner, which may significantly contribute to the complexity of recognition of toxins targeted to the ER translocons. One of the major, important chaperones that belong to this group, is calnexin (Cnx) and its soluble orthologue calreticulin (Crt). Both proteins recognize monoglucosylated glycans present on their protein substrates [126]. The asparagine residue of a consensus motif (Asn-Xxx-Ser/Thr or more rarely Asn-Xxx-Cys, Asn-Xxx-Val or Asn-Gly) of the nascent glycoproteins entering the ER is modified through the covalent attachment of an oligosaccharide core that is composed of two *N-*acetyl glucosamines, nine mannoses and three glucoses (Glc3Man9GlcNAc2) [127]. The glycoproteins then undergo trimming by glucosidases I and II (GI and GII), which sequentially remove two terminal glucose residues. Monoglucosylated, immature glycoproteins are bound by Cnx/Crt that are in complex with an oxidoreductase, ERp57 and attempt to obtain their proper structure [91,92]. After folding, glycoprotein is released from the Cnx/Crt cycle, what precedes removal of the final glucose from an oligosaccharide core by glucosidase II. This step creates an unglucosylated substrate and inhibits its rebinding to calnexin or calreticulin. At this stage, properly folded polypeptides can be transported to other cellular compartments, whereas unfolded proteins are retained in the ER and can be again recruited to the Cnx/Crt cycle [91,92]. Originally, it has been suggested that Cnx and Crt recognize their substrates exclusively via lectin-oligosaccharide biding sites [128,129]. Nowadays, there is evidence that other sites, including these for non-native polypeptides, are also crucial [130,131]. If the folding time is exceeded and the glycoprotein molecule cannot achieve its proper conformation or if the protein was extensively misfolded after it passed the Cnx/Crt cycle only once, it is finally targeted for ERAD. Different lectins participate in recognition of substrates to be targeted for proteasomal degradation or, similarly to toxins, for transportation to the cytosol to exert their cytotoxic functions. Among these chaperones, ER degradation-enhancing α-mannosidase-like proteins (EDEMs) seem to be the best described in relation to toxin recognition. EDEM family contains EDEM1, EDEM2 and EDEM3, that belong to the glycosyl hydrolase 47 (GH47) family [132,133,134,135]. This family also comprises ERManI and the Golgi α1,2 mannosidases [136]. It is still unclear exactly which signals and mechanisms regulate recognition of folding-defective polypeptides expressed in the ER, however, it seems that carbohydrate degradation signals (generated by removal of several mannose residues from the oligosaccharide core) play a crucial role in mammalian cells [137,138]. It has been demonstrated in vivo [134,139,140,141] and very recently also in vitro [142,143], that all EDEM proteins possess enzymatic α1,2-mannosidase activity. Besides glycan-dependent interactions, EDEMs can also bind protein substrates independently of the substrate glycosylation [144,145,146,147]. 

Ricin is the best studied toxin in the context of carbohydrate-binding chaperones and their role in ricin A chain transport from the ER to the cytosol and RTA toxicity. It has been demonstrated that this toxin is able to interact with calreticulin both, in vitro and in vivo [148]. That interaction occurred with the ricin holotoxin but not with a free ricin A chain; and it was prevented in the presence of lactose, indicating that it was mediated by the galactose-specific binding sites of the ricin B chain (Figure 4A). In addition, it was suggested that calreticulin is a candidate for a Golgi-to-ER recycling protein that might be opportunistically used by ricin to reach the ER lumen [148]. Ricin A chain can interact with EDEM1 [149,150], EDEM2 [150] and EDEM3 [151] (Figure 4A) but it seems that particular EDEMs can influence the RTA transport to the cytosol and its cytotoxicity quite differently. EDEM2 directly promotes RTA transport out of the ER, as high expression of this ER chaperone sensitizes cells to ricin [150]. Surprisingly, overexpression of EDEM1 decreases the RTA transport to the cytosol and protects cells against ricin [149,150]. However, the mode of EDEM1 action it not so obvious. It has been demonstrated that high expression of both, EDEM1 and EDEM2, promotes release of misfolded proteins from the Cnx/Crt cycle [132,152,153] and stimulates ERAD, what may decrease ricin transport through the translocon [149]. However, when EDEM1-transfected cells were treated with specific inhibitors that increase general accessibility of the ER translocons, much more ricin can be transported from the ER to the cytosol in comparison to the control cells. Additionally, in cells treated with these inhibitors, interactions between ricin and EDEM1 were significantly increased compared to the cells not treated with these inhibitors [149]. Experiments performed in cells transfected with siRNA against EDEM1 indicated a decreased transport of the ricin A chain to the cytosol. Thus, it was suggested that EDEM1 can increase RTA transport to the cytosol but only when ER channels become more accessible for ricin [149]. Interestingly, increased accessibility of ER translocons did not cause further acceleration of RTA transport to the cytosol in EDEM2-transfected cells [150]. This suggests that EDEM2-dependent retrotranslocation of RTA to the cytosol is not related to ER translocon accessibility. As was already mentioned, it has been demonstrated that EDEM1, EDEM2 and EDEM3 interact with the ricin A chain [149,150,151,154,155]. The nature of ricin interactions with EDEM proteins emerges as an important issue. Ricin A chain derived from plants contains two N-linked oligosaccharide chains [156]. However, in described experiments recombinant RTA that lacks oligosaccharides was used. Thus, such ricin should be perceived as a non-glycosylated ERAD substrate, confirming the significance of carbohydrate-independent interactions with EDEMs. Interestingly, higher level of ricin binding was found for EDEM2 than EDEM1 [150]. After analysis of the data that describe different translocon accessibility for ricin upon EDEM1 or EDEM2 overexpression, it was suggested that EDEM2 recognizes ricin and misfolded proteins in a similar way, whereas EDEM1 exhibits higher affinity to misfolded glycoproteins than to ricin. However, ricin carbohydrate-independent interactions with EDEMs cannot indicate that N-glycosylation of the A chain is completely negligible for RTA transport to the cytosol and its cytotoxicity. Experiments performed in *S. cerevisiae* demonstrated that glycosylation of RTA promotes its transport out of the ER [157]. This contributes to ricin toxicity, as lack of RTA glycosylation reduced cytotoxicity by impairing depurination of specific adenine in 28S rRNA [157]. Surprisingly, recently published results demonstrated that GFP-tagged RTA bearing a point mutation (E177Q) which attenuates its cytotoxicity (GFP-RTA E177Q) and engineered with a murine signal sequence for direct co-translational delivery into the host cell ER, was destabilized by disrupting genes required to generate and recognize the N-glycan residue [158]. These results suggest that the glycan signal that normally can promote degradation of misfolded glycoproteins in the ER actually stabilizes the GFP-RTA E177Q. Besides glycans present on RTA that might be recognized by lectin chaperones, it seems that the ricin A chain structure and appropriate hydrophobicity are important for its transport to the cytosol and overall cytotoxicity. The ricin A chain contains a highly hydrophobic, 12-amino-acid residue (Val245 to Val256) C-terminal region. This region is buried in the holotoxin but becomes exposed upon the A chain and B chain dissociation in the ER. Substitution of proline to alanine at amino acid position 250 (P250A) results in a significant decrease in modified ricin cytotoxicity in Vero (African green monkey kidney) and in HEK293 (human embryonic kidney) cells, as well as in reduced RTA P250A retrotranslocation to the cytosol [150,154,159]. It also appeared that the P250A mutation decreases interactions between RTA and EDEM1 and between RTA and EDEM2 [150,154]. Importantly, this mutation changes the RTA secondary structure to a more helical one [154]. Thus, ricin A chain interaction with EDEM1 and EDEM2 might be dependent on the appropriate structure of the toxin. Interestingly, translocation of modified RTA P250A from the ER to the cytosol, in contrast to wild-type RTA, appears to be independent on both, EDEM1 [154] and EDEM2 [150]. This might be connected with decreased interactions of RTA with EDEM1 and EDEM2. Moreover, it has been demonstrated that the C-terminal hydrophobic region of RTA is located at the ER membrane at the physiologically relevant temperature of 37 °C, before dislocation to the cytosol [160]. Insertion of the hydrophobic region into membranes might be possible due to the changes in the secondary structure of RTA which loses some α-helical structures. It cannot be excluded that RTA P250A, possessing an increased level of α-helices, is unable to undergo additional conformational changes allowing it to be stably exposed to the ER membrane. Another study also indicated that the C-terminal sequence of RTA is critical for RTA cytotoxicity [161] and ER exit [157]. It was shown that a double mutation in this region, P250L/A253V, eliminated depurination activity and cytotoxicity of RTA in yeast [161]. This was connected with inhibited transport of RTA P250L/A253V out of the ER [157]. However, not only the structure of RTA but also the degree of hydrophobicity, might be important for interactions with the ER chaperones [155]. It has been shown that a significant decrease in RTA binding to EDEM1 and EDEM2 may arise from reduced hydrophobicity of the RTA C-terminal region. On the other hand, further increase in hydrophobicity of this already highly hydrophobic region does not influence the interactions between RTA and EDEM1 and between RTA and EDEM2 [155]. These results indicate that for interactions between both, EDEM1 and RTA and EDEM2 and RTA, appropriate hydrophobicity of the substrate is crucial; too low hydrophobicity of the C-terminal region of RTA results in reduced interactions with EDEM chaperone proteins. 

In case of the cholera toxin, it is known that EDEM1 and OS-9 do not play a significant role in the CTA1 transport to the cytosol [106]. OS-9 is another lectin quality-control receptor that recognizes mannose-trimmed N-glycans [7,162] and additionally might be a Grp94 cofactor that helps in selection and targeting of the ERAD substrates [1,163].

## 3. Toxin Transport Across the ER Membrane

### 3.1. Putative ER Retrotranslocons

Several membrane proteins have been identified and proposed to form conducting channels present in the ER membrane and important in ERAD. These include the Sec61 complex [164,165,166,167,168], the Derlin proteins [12,13,169,170,171,172] and several ER-associated multi-spanning ubiquitin ligases, including HRD1 (Hrd1p in yeast) [21,173,174,175,176,177]. It is known that ubiquitin ligases are essential for the retrotranslocation of many substrates, not only for their potential involvement in the translocation process *per se*, but they are also responsible for adding polyubiquitin chains to polypeptides emerging in the cytosol during retrotranslocation. Polyubiquitination is required for subsequent extraction of ERAD substrates by the VCP/p97 ATPase (Cdc48 in yeast) and its cofactors (Ufd1-Npl4) and their further recognition by the 26S proteasome [21,178,179,180,181]. Toxins are not typical ERAD substrates, they are not transported to the cytosol for proteasomal degradation; instead, toxin translocation out of the ER becomes part of their intoxication route. Data presented so far indicate that toxins can generally use the same ER translocons as misfolded proteins in their transport to the cytosol, however, similarly to ERAD substrates, this process is very elaborated.

A single Sec61-complex functions as a protein-conducting channel. Its structure is highly conserved and consists of a heterotrimer of Sec61α, -β and -γ, where Sec61α is the largest, major transmembrane component that spans the membrane ten times [182,183,184]. Sec61β and Sec61γ are single spanning membrane proteins belonging to the family of tail-anchored proteins. Sec61 is undoubtedly the main translocon involved in co-translational protein transport into the ER [183,185,186]. The contribution of Sec61 to substrate dislocation during ERAD in mammalian cells is not clear and is still under debate. Some data, also describing toxins or viruses that utilize ERAD, demonstrate that Sec61 may be involved in retrograde transport to the cytosol [164,165,166,167,168,187]. The discovery of export-specific *sec61* mutants in yeast [166] and indication that interaction of the proteasome 19S regulatory particle (RP) with the Sec61 channel is essential for the export of specific substrates to the cytosol for proteasomal degradation [167], suggest that denying Sec61 role in ERAD may have been premature [168]. Moreover, it was suggested that the import of nascent proteins into the ER and dislocation of aberrant proteins from the ER is connected with two activities of yeast Sec61p (Sec61α in mammals) that are mechanistically different because they involve distinct domains within Sec61p [188]. Dislocation-defective mutants of Sec61p were still proficient in protein import into the ER. Very recently, it has been also demonstrated that N-terminal acetylation of Sec61p plays a role in ERAD [189]. On the other hand, it is concluded that mutations in *sec61* gene may indirectly alter the biosynthesis of important ERAD components [10,174]. Moreover, the Sec61 channel is plugged by some kind of gating proteins (most probably BiP and TRAP), which are displaced by a signal sequence of a secretory protein during co-translational translocation into the ER [190,191,192]. Therefore, it is debated how the channel would open from the luminal side of the ER during ERAD [10]. Finally, some results directly demonstrate that Sec61 in not involved in the ER-cytosol transport, showing for example, that Sec61 blockade by mycolactone does not inhibit retrotranslocation of the ERAD substrates [193].

The initial suggestion that Hrd1p can form an ER channel important in ERAD comes from experiments performed in yeast, indicating that Hrd3p and Der3p/Hrd1p are constituents of a highly dynamic complex organized around the Sec61 pore [194]. As was already mentioned, in *S. cerevisiae*, substrates use three ERAD pathways (ERAD-L, -M and -C), depending on whether their misfolded domain is located in the ER lumen, ER membrane or on the cytoplasmic side of the ER membrane [8,195]. Further evidence that Hrd1p forms retrotranslocation channel was supported by experiments showing that this protein is the central membrane component in the ERAD-L process [174]. Currently, it is believed that Hrd1p can function both, in the ERAD-L and –M [9,10]. In yeast, substrates need to be bound to the membrane-embedded domain of Hrd1p to become polyubiquitinated [175], moreover auto-ubiquitination of Hrd1p was postulated to be the trigger for retrotranslocation of the substrate to the cytosol. Current model describes auto-ubiquitination of Hrd1p as a factor that opens the channel for ERAD-L substrates [10,175,176]. The SEL1L-HRD1 protein complex represents the most conserved ERAD machinery in mammals, with SEL1L being the cofactor for the E3 ligase HRD1 [21,169,173,196,197]. SEL1L is absolutely required for the stability of HRD1 [198] and may directly interact with and recruit substrates to the HRD1 channel [197,198].

Another protein that could form a channel important in ERAD is Derlin-1, a mammalian homolog of yeast Der1 [12,13]. This is a multispanning membrane protein that has interaction partners on both sides of the ER membrane. Derlin-1 promotes retrotranslocation of MHC class I heavy chains from the ER to the cytosol [12,13]. However, this protein is also involved in extraction of certain aberrantly folded proteins from the ER [199,200,201,202]. It was also shown that the p97 ATPase can be recruited to Derlin-1 by interactions with VIMP (valosin-containing protein-interacting membrane protein), to facilitate protein retrotranslocation from the ER lumen to the cytoplasm for degradation by the 26S proteasome [12,203]. Mammalian genomes encode two additional, related proteins: Derlin-2 and Derlin-3, that span the ER membrane multiple times [169,170]. It has been demonstrated that they are also required for ERAD of misfolded glycoproteins [169,170,204]. EDEM1 can interact with Derlin-2 and Derlin-3 [170]. Interestingly, overexpression of Derlin-2 facilitates association of EDEM1 with a cytosolic ATPase p97. On the other hand, it should be considered that the Derlin proteins do not form a separate translocon but initiate the export of aberrant polypeptides from the ER lumen by threading them into the ER membrane and routing ERAD substrates to other ER channels. It was indicated that Derlin-2 and Derlin-3 co-localize with Sec61β, a component of Sec61 translocon [170]. It was also postulated that Derlin-2 may regulate the movement of substrates through the HRD1 retrotranslocon [205]. In yeast, Der1 is a membrane protein of the Hrd1p complex, involved in ERAD-M and ERAD-L [9,206]. Finally, it was even suggested that instead of binding to the unfolded ERAD substrates in the membrane as they pass into the cytoplasm, Derlins simply regulate the activities of other integral membrane components of the ERAD machinery [207]. 

### 3.2. Dependence of Toxin A Chain Transport to the Cytosol on the ER Translocon Complexes 

Strong evidence that the Sec61 complex can be used in transport from the ER to the cytosol comes from experiments in which transfer of the cholera toxin CTA1 subunit to the cytosol was reconstituted in a cell-free system, using ER-derived translocation-competent microsomes [112]. It was demonstrated that CTA1 interacts with Sec61p (Figure 3A) and moreover, when the Sec61p complexes were blocked by nascent polypeptides arrested during import, export of CTA1 was inhibited [112]. However, transport of the cholera toxin A subunit trough the ER membrane does not seem to be so simple, as Derlin-1 [104,109], HRD1 and SEL1L [106,208,209] also facilitate translocation of CTA1 to the cytosol (Figure 3A). Overexpression of a dominant-negative Derlin-1-YFP decreased the ER-to-cytosol transport of CTA1. Co-immunoprecipitation studies demonstrated that Derlin-1-YFP associates with CTB, CTA and PDI but significant interactions were not detected between CTB and Derlin-2 [109]. It was suggested that the dominant-negative Derlin-1 exerts its inhibitory action by titrating CT from Derlin-1 and inducing a structural defect on Derlin-1. Dominant-negative Derlin constructs (Derlin-1-GFP and Derlin-2-GFP) have been characterized before [12], and in the work just described [109] YFP constructs were used instead of GFP. However, it should be noted that several years after the Derlin-YFP constructs were used to study CTA1 transport to the cytosol, it was evaluated that Derl2-GFP was unable to bind the AAA ATPase p97 [43]. Surprisingly, further studies revealed that despite failing to interact with p97, Derl2-GFP did not act as a dominant negative inhibitor. If this is also the case for Derlin-1-YFP constructs, it would mean that the Derlin-1-p97 interactions are especially crucial for CTA1 transport to the cytosol. Two independent reports suggest that p97 plays a role in facilitating CTA1 retrotranslocation [210,211] and there is evidence that the cholera toxin A chain interacts with endogenous p97 [210] (Figure 3A). The importance of Derlin-1 in CTA transport to the cytosol was further indicated in experiments which demonstrated that suppressing Derlin-1 with siRNA protected cells from cholera intoxication [104]. In addition, Derlin-1 co-immunoprecipitated with CTA or CTB from CT-treated cells. However, the cholera toxin itself might up-regulate ERAD proteins that sensitize cells to the toxin, as it was found that the levels of BiP, Derlin-1 and Derlin-2 quickly increased upon exposing cells to CT or CTB [104]. Thus, experiments in which the Derlin-1 levels were altered during CT challenge in order to verify the role of Derlin-1 in CTA1 transport to the cytosol, would not be so simple to interpret. It should be mentioned that experiments in which zebrafish was established as a genetic model for the study of the mechanisms of cholera intoxication, revealed that both, Derlin-1 and -2 are dispensable for retrotranslocation of the CTA1 [212]. The role of E3 ubiquitin ligases in CTA1 transport to the cytosol was initially unclear, since CTA1 is neither ubiquitinated on its lysine residues nor at its N-terminus. However, it was demonstrated that the HRD1 and gp78 ligases are important members of the cholera toxin retrotranslocation machinery [208] (Figure 3A). The usage of HRD1 and gp78 mutated versions, as well as a *HRD1* knockdown, have led to a block in toxin translocation. Co-immunoprecipitation analyses demonstrated that HRD1 and gp78 bind to CTA, CTB and PDI. In addition, the binding studies also indicated sequential transfer of the toxin from Derlin-1 to E3 ligases before exiting the ER [208]. However, the cholera toxin retrotranslocation complex is more intricate. The HRD1 adaptor, SEL1L, also binds CTA and facilitates toxin retrotranslocation [106] (Figure 3A). Importantly, ERdj5 (ER-localized Hsp40, already described in this review) interacts with SEL1L directly through its N-terminal lumenal domain, thereby linking ERdj5 to the HRD1 complex [106]. Considering the fact that CTA can be transferred from ERdj5 to BiP [213], because ERdj5 binds to BiP and regulates the BiP–CTA interaction [106], it can be assumed that BiP–toxin interaction occurs proximally to the HRD1 complex. Thus, the model in which HRD1 retrotranslocation machinery captures the cholera toxin once it is released from BiP is highly probable (Figure 3A). 

The ricin A chain transport to the cytosol has been intensively studied for a long time, however it seems that the more is known, the more questions appear. For many years it has been considered that Sec61 can be utilized by the ricin A chain in its transport from the ER to the cytosol [214]. Experiments performed in yeast clearly demonstrated that pulse-labelled RTA was stabilized in *sec61* mutant strains. The conviction of the Sec61 complex involvement in the RTA retrotranslocation was also strengthened by observations that RTA can interact with Sec61α [123,149] (Figure 4A). However, when genome-wide RNAi screens were employed to identify genes required for ricin intoxication, it appeared that ricin toxicity does not depend on Sec61 [215]. This was estimated in experiments in which *Sec61* knockdown was obtained by using a mix of the single most potent siRNAs against each gene of the Sec61 complex. In agreement with these results, direct retrotranslocation assays performed in HEK293 cells, in which the level of Sec61α was significantly downregulated, did not indicate that transport of RTA from the ER to the cytosol depends on Sec61α [216]. These results have to be further analysed and it cannot be excluded that upon Sec61α downregulation, other ER channels can be utilized by RTA which would compensate for the inhibition of RTA transport by the Sec61 complex. Studies carried out in both, the yeast and mammalian cells, suggest a role for HRD1-SEL1L complex in RTA retrotranslocation (Figure 4A). In yeast, RTA variants (native and misfolded) were expressed in the ER lumen by targeting the nascent proteins with a Kar2 signal peptide [217]. Both forms of RTA require the Hrd1-Hrd3p complex for export from the ER. Moreover, null strains lacking the Hrd1p cofactors: Hrd3p (SEL1L in mammals), Der1p (Derlin-1 in mammals) or Usa1p (Herp in mammals) (Figure 4A), exhibit phenotypes similar to the ∆*hrd1* strain [213,217], in which the rate of RTA transport to the cytosol was reduced. In mammalian cells, it has been demonstrated that ricin requires SEL1L for the dislocation of its A chain from the ER to the cytosol [218] (Figure 4A). Consistent with these results, *SEL1L* knockdown protects cells from ricin [218]. Cells stably transfected with dominant negative Derlin-1 and Derlin-2 constructs and treated with purified ricin did not reveal a change in ricin A chain transport to the cytosol when compared to the control cells [149]. Similar results were obtained for dominant negative Derlin-1 in transfected mammalian cells expressing an ER-localized RTA construct [218]. However, considering observations that dominant-negative Derlin constructs solely inhibit the Derlins-p97 interactions [43], perhaps it should be concluded that Derlin interactions with p97 are dispensable for RTA transport to the cytosol.On the other hand, overproduction of unmodified Derlin-1 and Derlin-2, also did not influence the ricin A chain dislocation [219] Moreover, it has been demonstrated that a ∆*der1* yeast strain does not display a defect in the ERAD processing of ectopically expressed, ER-localized RTA [214]. Interestingly, Derlin-1 is required for an efficient retrograde transport of ricin from endosomes to the Golgi apparatus [220]. This effect was attributed to observed slight resistance to ricin obtained in cells with reduced level of the Derlin family proteins [220].However, a mix of siRNAs against the three Derlins resulted in a significant rescue of ricin toxicity [215] (Figure 4A). This effect was assigned to the ricin A chain ER-cytosol transport. In addition, identification of UFD1L and NPLOC4 as ricin specific factors was consistent with the role of Derlins in ricin translocation across the ER membrane [215] (Figure 4A). It has been demonstrated that these two factors form a ternary complex with the p97 ATPase which is required for protein export from the ER and has been shown to bind to Derlins [170]. Data presented in later studies indicated that Derl2 and Hrd1p contribute to but are not required for sensitivity to ricin [43]. 

*Pseudomonas* exotoxin A (PE) toxicity has been also analysed by using siRNA pools comprising the best individual siRNA for each gene of the Sec61 complex or the Derlins [215]. Downregulation of the Sec61 complex resulted in a strong and specific rescue of this toxin (Figure 3B), whereas a mix of siRNAs against the three Derlins did not change the PE intoxication [215]. These results are in agreement with earlier experiments. It was demonstrated that *Pseudomonas* exotoxin A can be co-immunoprecipitated with Sec61α [221] (Figure 3B). The interactions between PE and the Sec61p translocon in ER-derived microsomes blocked transport of immunogenic peptides from the ER to the cytosol [221]. Moreover, it was shown that the N-terminus of Sec61α subunit is the relevant binding site for PE [222]. Exogenously applied *Pseudomonas* exotoxin A can also inhibit passive Ca^2+^ leakage from the ER through the Sec61 pore [222]. 

Interactions between Shiga toxin StxA1 subunit and Sec61 have been also demonstrated [119] (Figure 4B). It was shown that a significant amount of HEDJ (ERdj3)-bound toxin was associated with Sec61 (Figure 4B). These data may suggest that Stx is recruited to the Sec61 apparatus by HEDJ and other luminal ER chaperones [119]. However, to fully evaluate the role of Sec61 in StxA1 retrotranslocation, functional interactions between the Shiga toxin A chain and the translocon should be studied. In contrast, experiments performed in yeast have demonstrated a fully functional role of Hrd1p in StxA1 transport from the ER to the cytosol [223] (Figure 4B). StxA1 was more toxic in wild-type strains that in the ∆*hrd1*. Interestingly, contrary to RTA, catalytic activity of Hrd1p was required in StxA1 retrotranslocation. However, canonical ubiquitination was not necessary for dislocation of the toxic fraction of Shiga-like toxin, SLTxA1 [223]. Currently, there are no studies performed with the yeast Sec61p mutants. Thus, it remains unsettled if both, Sec61 and HRD1, can be used in the yeast and mammalian cells or if StxA1 uses distinct translocons in yeast and mammals [213]. It is also possible that the Sec61 complex does not serve as a StxA1 translocon, since only interactions between Sec61 and StxA1 were reported in mammalian cells, without demonstrating the role of Sec61 in StxA1 transport to the cytosol [119]. 

The mechanism by which cytolethal distending toxins (CDTs) exit the ER is very poorly known. It should be noted that several reports have suggested that ERAD does not play a role in the translocation of CDT across the ER membrane [57,87]. However, results published by the Kenneth Bradley group indicate that three important components of the retrotranslocation machinery, Derlin-2 (Derl2), the E3 ubiquitin-protein ligase HRD1 and the AAA ATPase p97, are required for intoxication by some CDTs [43] (Figure 4C). In addition, two previously uncharacterized functional domains in Derl2, the N-terminal 88 amino acids and the second ER-luminal loop were identified as crucial for intoxication by CDT encoded by *Haemophilus ducreyi* (HdCDT).

## 4. Toxin Extraction from the ER Membrane, Refolding and Activation in the Cytosol

The majority of exported ERAD substrates are degraded by the cytosolic ubiquitin-proteasome system (UPS). In this system, the substrates for degradation are first polyubiquitinated via an enzymatic cascade involving E1 Ub-activating enzyme(s), E2 Ub-conjugating enzyme(s) and E3 Ub ligases [224]. It was established that three classes of E3s operate in ERAD: (1) RING domain, (2) HECT domain and (3) U-box domain E3s. RING domain and U-box domain E3s promote transfer of ubiquitin from the E2 ubiquitin-conjugating enzyme to the substrate, whereas the HECT domain E3s forms a thiol ester with Ub before transferring it to the substrate lysine [225,226]. However, it should be noted that auto-ubiquitination of the Hrd1p RING domain is postulated to be crucial for initiating retrotranslocation of the substrate [176]. After an ERAD substrate has been adequately polyubiquitinated, the p97/Cdc48 complex is recruited to the substrate by p97 cofactors, Ufd1/Npl4 heterodimeric complex and in some cases Ubx2p (UBXD8 in mammalian cells) [227,228,229]. p97 has been found to be involved in a large variety of cellular processes, with a crucial role in ERAD [230]. The Ufd1/Npl4 complex possesses both, the ubiquitin-binding domains and p97-interacting motifs [231,232,233,234]. It has been demonstrated that human HRD1, as well as gp78, associate with p97 and the ER-membrane protein Derlin-1 [235,236,237]. Association of Derlin-1 with p97 was shown to be independent of HRD1, indicating that at least two distinct protein complexes containing p97 operate at different steps of protein dislocation [169,236,238]. In yeast, Ubx2p, an ER-membrane protein, was found to confer interaction between the Cdc48p complex and the ubiquitin ligases Hrd1p and Doa10p [228,229]. The p97/Cdc48p AAA-ATPase is the main driving force that pulls ERAD substrates to the cytosol. Then, the proteins are recognized and turned over by the 26S proteasome, which is a large multi-subunit complex of proteases and regulatory proteins. It is formed by two complexes: a barrel-shaped proteolytic 20S particle and a 19S cap which is located at one or both ends of the 20S particle [239]. It was proposed that in certain degradation pathways, Dsk2p, Rad23p and the trimeric Cdc48 form a complex that functions together in the delivery of ubiquitinated proteins to the proteasome [240]. Once Rad23 and Dsk2 bind the Cdc48 complex, they link the ERAD substrate to the proteasome through a component called Rpn1, which resides on the 19S proteasome particle [241]. Another component of the 19S cap, Rpn10, can bind ubiquitin in a Rad23-independent manner [242]. After binding of the substrate to the 19S particle, the proteasome-associated deubiquitinating enzymes (DUBs), Ubp6 and Rpn11, cleave the ubiquitin chain and the deubiquitinated protein is subsequently directed to the 20S core particle and degraded [243]. 

Both, the p97-dependent and independent pathways are utilized by various ERAD-related toxins. Since the A subunits of ERAD-dependent toxins exert their cytotoxic activity in the cytosol, it is assumed that at least partially they must avoid polyubiquitination and proteasomal degradation. However, there are also examples of ubiquitin-independent proteasomal degradation [244,245]. This phenomenon can be explained by an observation that the A subunits of ERAD-dependent toxins have an extreme arginine-over-lysine amino acid bias that is not found either in the B chains of these toxins [246] or in the A subunit of toxins that are trafficked by other pathways (such as the diphtheria toxin) [247]. It was suggested that the lack of lysine residues is a mechanism for escaping ubiquitin-mediated protein degradation during translocation of ER-directed AB-toxins into the cytosol [248]. Moreover, it has been demonstrated that the isolated A subunits resemble unfolded proteins and in some cases the hydrophobic C-terminal domain contributes to the A subunit unfolding via an interaction with the ER membrane [213]. After transport to the cytosol, the A subunit of ERAD-related toxins, in contrast to misfolded proteasome directed proteins, must refold to obtain its active conformation. Different host factors and chaperones are involved in this process. 

It has been demonstrated that CTA1 of the cholera toxin is not ubiquitinated at both, its two lysine residues and its N-terminus and that CTA1 retrotranslocation to the cytosol does not depend on polyubiquitination or the proteasome function [248]. It was concluded that the reason why the toxin escapes degradation in the cytosol may be attributed to both, its scarcity of lysines and its rapid refolding. However, the ubiquitin ligase activity of HRD1 and/or gp78 is necessary for dislocation of CTA1 [208]. It cannot be excluded that CTA1 is ubiquitinated on non-lysine residues, which is probably rare but is possible [249] or that ubiquitination of other cellular factors may be required to pull CTA1 to the cytosol [250]. This second hypothesis is much more probable as it was shown that in the mutated toxin with the N-terminally extended A1 chain, two lysine residues present at native CTA1 (Lys-4 and Lys-17) become ubiquitinated, which has caused a rapid degradation of modified CTA1 [251]. Thus, retrotranslocation of the wild-type toxin must proceed in a way that protects these lysine residues from attack by E3 ligases. It has been shown that CTA1 can interact with p97 (Figure 3A) and that expression of dominant p97 mutant slightly inhibits the cytotoxicity of CT and increases the time required for CT delivery to the cytosol [210]. However, other studies suggested that p97 does not provide the primary driving force for extracting the A1 chain from the ER, which is consistent with a requirement for polyubiquitination in p97 function [211]. To clarify cytosolic events controlling CTA1 release from the ER into the cytosol, an in vitro assay in semi-permeabilized cells was developed. It was demonstrated that CTA1 is released into the cytosol as a folded molecule in a p97- and proteasome-independent manner. Such release nonetheless involves a GTP-dependent reaction [252]. Interestingly, Ufd1-Npl4 complex acts as a negative regulator of the cholera toxin retrotranslocation, in this case acting independently of p97 [253]. It was also demonstrated that deubiquitinase YOD1 negatively controls CTA1 retrotranslocation, likely by deubiquitinating and inactivating ubiquitinated ERAD components that promote toxin retrotranslocation [254]. Interestingly, cytosolic Hsp90 is required for CTA1 dislocation to the cytosol [116] (Figure 3A). This contribution is significant since cells with reduced levels of Hsp90 were highly resistant to the CT intoxication. Hsp90 could bind to CTA1 at 37 °C in an ATP-dependent manner [116]. At this temperature, the A subunit is in an unfolded conformation [66,67], which suggested that Hsp90 recognizes an unfolded conformation of CTA1 during the dislocation event. In addition, it was shown that ATP hydrolysis by Hsp90 is required for CTA1 extraction from the ER [255]. Hsp90 would prevent the unfolded CTA1 protein from sliding back into the dislocation pore by coupling dislocation with refolding. This may resemble a ratchet mechanism that would provide the driving force for CTA1 dislocation. [116,255]. In vitro, a CTA1 with disturbed conformation can be degraded in a ubiquitin-independent manner by a core 20S proteasome [66] (Figure 3A). Degradation did not occur when CTA1 tertiary structure was stabilized [67]. This stabilization also blocked retrotranslocation. Thus, in vivo, conformational instability of CTA1 promotes degradation by proteasomal 20S particle [66,213]. However, a portion of CTA1 is not degraded and may exert its cytopathic action [213,248,251]. The refolding of translocated CTA1 to an active conformation appears to be a complex process that involves a sequential interaction of CTA1 with Hsp90 and lipid rafts [116,255,256] (Figure 3A). Hsp90 binds CTA1 with very high affinity and it is not released after CTA1 refolding [116,255]. It was proved that this chaperone can convert disordered CTA1 to a structured conformation [255]. Moreover, the CTA1 and Hsp90 complex could bind to lipid rafts (Figure 3A). Lipid rafts exhibit a chaperone-like function that returns disordered CTA1 to an active state and is required for the optimal in vivo activity of CTA1 [256]. In addition, the C-terminal end of CTA1 seems to facilitate toxin-lipid interactions that promote either toxin unfolding in the ER or toxin refolding in the cytosol. It appears that a similar model exists for the ricin and Shiga toxin A and A1 chains, respectively. 

The ricin A chain contains only two lysine residues which generally do not become targets for ubiquitination in mammalian cells during toxin dislocation [76,257,258]. However, a low level of cytosolic ubiquitination of RTA does occur via an unknown E3 ligase [217]. Introduction of additional lysine residues into RTA reduces its cytotoxicity by increasing the extent of ubiquitin-mediated proteasomal degradation [76]. Similarly, experiments performed in tobacco protoplasts had shown that accumulation of active toxin in the plant cytosol is exquisitely sensitive to lysine content, more so than in the mammalian cytosol [258]. Experiments performed in yeast demonstrated that transport of the ricin A chain to the cytosol is not driven by the ubiquitin-dependent system, Cdc48p [217] (Figure 4A). However, ectopic co-expression of RTA in the ER of tobacco protoplasts with a dominant negative Cdc48, clearly demonstrated that retrotranslocation of the ricin A chain requires participation of Cdc48 and is independent of its glycosylation or ubiquitination status [259]. Moreover, expression of dominant negative p97 mutant in mammalian cells inhibited ricin toxicity and increased the time required for RTA transport to the cytosol [210]. Thus, the ricin A chain appears to utilize a p97/Cdc48-dependent but ubiquitin-independent, extraction mechanism [213] (Figure 4A). Interestingly, transport of RTA to the cytosol is also mediated by an ATPase subunit of the 19S proteasome cap, Rpt4p [217] and two other proteins of that cap, Cim3p and Cip5p [214] (Figure 4A). However, it shows no obvious requirement for the other Rpt subunits, Ubr1p or the proteasome core itself [217]. It should be mentioned that Rpt4p can act with Cdc48p in extraction of an endogenous substrate from the yeast ER [260]. Thus, similar mechanism may operate during RTA transport to the cytosol. It is known that ricin is partially degraded by proteasomes in mammalian (Figure 4A) and plant cells and that this degradation can be inhibited by proteasome inhibitors [123,149,150,154,261]. Data derived from studies in yeast had shown that a *pre1-1* mutant that is deficient in the chymotrypsin-like activity of the proteasomal core, does not display any significant defects in the turnover of RTA [217,262]. However, pulse-chase experiments performed in yeast demonstrated that most of the RTA was degraded during the first hour of the chase but around 20% was not degraded and appeared to be completely stable. Cell fractionation had shown that this stable RTA was in the soluble, rather than the microsomal fraction, indicating that RTA was present in the cytosol [214]. Interestingly, similarly to EDEM proteins acting in mammalian cells [154], yeast proteasome can discriminate between structural features of the same substrate [217]. A structurally defective form of RTA was much more susceptible to proteasomal degradation than native RTA. Generally, acquisition of catalytic conformation by RTA could be obtained by: ribosome-mediated refolding [83], spontaneous refolding [248] and interactions with cytosolic chaperones [125] and cytosolic factors [262]. The Rpt5p subunit of the 19S proteasome cap prevents aggregation of denatured RTA [262]. Furthermore, in vivo, the ATPase activity of Rpt5p is required for maximum toxicity of RTA dislocated from the yeast ER. Inhibition of cytosolic Hsc70 protected from, whereas inhibition of Hsp90 sensitized cells to ricin [125]. It was proven that RTA interacts with Hsc70 in vitro (Figure 4A), which prevents aggregation of the heat-treated toxin. In addition, RTA catalytic activity was recovered after chaperone treatment. Importantly, the co-chaperone activity balance which regulates Hsc70 and Hsp90, functions to determine the fate of dislocated RTA. Sequential interaction with Hsc70 and Hsp90 directs cytosolic RTA toward net inactivation [125] (Figure 4A). In other words, a network of chaperones regulates the competing processes of folding and degradation. In yeast, no clear growth advantage was seen for cells lacking individual Hsp40, Hsp70 and Hsp90 family members or the Hsp70 and Hsp90 co-chaperones [217].

StxA1 of the Shiga toxin, similarly to ricin, has only two lysine residues, however, it seems that the bulk fractions of the toxin are polyubiquitinated [223]. On the other hand, toxin ubiquitination was not a general requirement for export from the ER since a variant of Shiga-like toxin, SLTxA1, possessing less lysine residues could still exit the ER to generate a cytotoxic effect [223]. Cdc48p is involved in SLTxA1 extraction from the ER (Figure 4B). The toxin subunit was also stabilized in a yeast strain lacking Npl4p [223]. Moreover, SLTxA1 was very strongly stabilized in absence of the proteasomal receptor Rad23p (Figure 4B) and increased stability of SLTxA1 was observed in a *pre2-2* yeast strain that is mutated in the β5 subunit of the 20S proteasome [223]. These data demonstrate that a part of SLTxA1 population is degraded by the proteasome (Figure 4B). Consistently, lactacystin, a proteasome inhibitor, increased cytosolic SLTxA1 by 30% and enhanced SLTxA1 cytotoxicity due to a 50% increase in overall protein synthesis inhibition [85]. Thus, ER extraction of some SLTxA1 is carried by an ERAD-enabled Cdc48p complex (Figure 4B). The C-terminal region of SLTxA1 contains a relatively hydrophobic stretch of amino acids that is important for cytotoxicity [263]. Peptides based on this region interact with lipid membranes at low pH, possibly insert into the ER membrane at neutral pH [264], which may allow the toxin subunit to be recognized by the ER quality control surveillance, similarly to misfolded proteins. Interestingly, the toxic fraction of SLTxA1 is also extracted as ubiquitinated proteins (Figure 4B). Presumably, this toxin fraction would need to be stripped of polyubiquitin chains to allow refolding to a functional conformation, which would suggest an intervention of a deubiquitylase (Figure 4B). Recovery of the toxin activity did not depend on a group of Cdc48p co-factors. It was suggested that a portion of SLTxA1 evades the cytosolic Cdc48p complex and subsequent targeting to the proteasome core, allowing uncoupling from ERAD and the expression of toxin activity in the cytosol. The Otu1p deubiquitylase and the substrate release factor Vms1p were proposed to act upstream of Cdc48 interactions [223] (Figure 4B). It was suggested that Hsp90 is not required for StxA1 transport to the cytosol, since geldanamycin, a Hsp90 inhibitor, did not affect the Stx activity against cultured cells [213]. 

PTS1 of the pertussis toxin is deprived of lysine residues. Construction of different lysine variant toxins demonstrated that these toxins had reduced cellular activity, which can be restored in the presence of a proteasome inhibitor [227]. PTS1 variants with more than one lysine change had a significantly greater cellular activity when the host cells were pre-treated with a proteasome inhibitor. These observations indicate that wild-type PTS1 avoids proteasomal degradation due to an absence of lysine residues [227]. Experiments in which surface plasmon resonance system [265] was applied to quantify the cytosolic pool of PTS1 in intoxicated cells, demonstrated that cells treated with a proteasome inhibitor contained larger quantities of cytosolic PTS1 [75]. This suggests a role for ubiquitin-independent proteasomal degradation in the PT intoxication process (Figure 3C). The quantity of cytosolic PTS1 appears to represent a balance between toxin delivery to the cytosol and toxin degradation in the cytosol. These observations are consistent with earlier experiments showing that the PTS1 subunit is a thermally unstable protein which can be degraded by the core 20S proteasome in a ubiquitin-independent fashion [74]. A disordered form of PTS1 subunit is probably crucial in this process, since PTB pentamer or holotoxin associated PTS1 were resistant to 20S proteasome degradation [74]. PTS1 is stabilized in the cytosol by NAD [74] (Figure 3C), the donor molecule for the ADP-ribosylation reaction that modifies the G protein targets of PT. NAD prevented PTS1 transition to a protease-sensitive conformation. Interestingly, this effect was not observed for the cholera toxin CTA1, which also uses NAD as the donor molecule [74]. 

Little is known about the ER membrane extraction and cytosolic host factor requirements in the case of other ERAD-dependent toxins. For *Pseudomonas aeruginosa* exotoxin A, expression of a dominant negative p97 mutant in mammalian cells inhibited PE toxicity and increased the time required for the A subunit transport to the cytosol [210] (Figure 3B). *C*ytolethal distending toxins possess the arginine-over-lysine amino acid bias present in their A subunits. On the other hand, the *Haemophilus ducreyi* A chain, HdCdtB, is resistant to degradation by the 20S proteasome in vitro [87]. Cell-based assays further suggested that HdCdtB does not unfold before exiting the ER and that it may move directly from the ER lumen to the nucleoplasm. As was already mentioned, the interactions between Derl2 and p97 are not required for HdCdtB retrotranslocation [43]. Expression of a dominant negative p97 caused a reduction in cell cycle arrest in G2, mediated by HdCDT, when compared to control p97. This resulted from retention of HdCDT in the ER [43]. How and whether nuclear translocation of CdtB takes place is still an open question (Figure 4C). As was already mentioned, some data point that CDT are transported from the ER to the nucleus. These data demonstrated that CdtB was localized within the ER membrane, close to the nucleus (i.e., nucleoplasmic reticulum) and was not found in the cytoplasm.This may suggest the model in which CDTs are translocated directly from the ER lumen into the nucleoplasm [87] (Figure 4C). Contrary to these data, other results have indicated requirements for nuclear localization signals (NLSs) within the CdtB subunits, suggesting that CdtBs are retrotranslocated to the cytosol prior their transport to the nucleus [266,267,268]. It is possible that both models exist and are specific for particular CDTs (Figure 4C). 

## 5. Concluding Remarks 

More than 20 years ago it was suggested that several AB-toxins can subvert the ERAD pathway to enter their target cells [23]. Since then, a huge progress has been made in understanding the mechanisms and cellular factors regulating ERAD, as well as the toxin intracellular transport and their biology. However, these two scopes were not developed independently, they were connected with each other in many aspects. This happened because toxin transport from the ER to the cytosol by ERAD is a crucial part of toxin intracellular routing that significantly contributes to regulation of their toxicity. On the other hand, toxins are good models of ERAD substrates. Thus, they have contributed to the general ERAD knowledge and general understanding of cell biology. This knowledge might be directly related to some medical aspects. As an example, toxins compete for ERAD with endogenous misfolded proteins, that for some reason should not be degraded. Therefore, ERAD-derived toxins could be used directly in therapy of certain genetic protein misfolding diseases. In ERAD-dependent genetic diseases mutant proteins are targeted to the ER translocons, retrotranslocated to the cytosol and prematurely degraded by the proteasome [269,270]. It was assumed that inhibition of ERAD could partially improve impaired intracellular transport and positively affect subcellular localization and enzymatic activities of mutated enzymes. Several ERAD inhibitors have been developed [196,271] and tested for their therapeutic effects [272,273]. However, accumulation of misfolded proteins can induce the unfolded protein response and lead to apoptosis [91,274]. Thus, such ERAD inhibitors can be relatively safely and efficiently used against cancer [275,276] but become problematic in the treatment of protein misfolding diseases. Recently, it has been demonstrated that genetically modified, catalytically inactive A subunits of the cholera or Shiga toxins compete for ERAD to rescue endogenous misfolded proteins from premature degradation [277]. Both A subunits reduce degradation of F508del CFTR, the major mutant protein responsible for cystic fibrosis and N370S glucocerebrosidase (GCC) causing the Gaucher glucosylceramide lysosomal storage disease [278]. It was suggested that such toxins could provide a new, general and competitive means to temporarily reduce the transit of endogenous ERAD substrates into the cytosol for degradation. Similar mechanism was also observed for the *Pseudomonas* exotoxin A and Sec61p translocon [221]. As was already mentioned, the interactions between PE and the Sec61p in ER-derived microsomes blocked transport of immunogenic peptides from the ER to the cytosol [221]. These experiments were based on the assumption that the A subunit of toxins and ERAD substrates utilize the same/similar translocon machinery for ER exit and that theoretically only one protein can occupy the translocon at a time. However, it seems that prevalence of the toxin A subunits over the misfolded proteins in the ER channel occupation does not always operate in the same way. Previously, it was shown that overproduction of model misfolded proteins (β-site amyloid precursor protein cleaving enzyme isoform, membrane BACE 457, luminal BACE 457 [152] and α1-antitrypsin (A1AT) null variant (Hong Kong, NHK; [132])) inhibited retrotranslocation of the ricin A chain from the ER to the cytosol [149]. In this manner, an increased transport of the misfolded proteins could occupy channels otherwise used for retrotranslocation of the ricin A chain. In agreement with these results, when the Sec61p complexes were blocked by nascent polypeptides arrested during import, export of the cholera toxin CTA1 was inhibited [112]. This suggests that in experiments described above, dislocation of toxins through the ER channels may depend on the type and perhaps amount of misfolded proteins that occupy the ER and/or it is highly probable that this transport is toxin specific. Future studies are necessary in order to estimate relations between the toxin and misfolded protein transport during ERAD. Such studies will undoubtedly contribute to the general knowledge that will be useful in medical applications. 

In the end, it should be noted that studies of protein toxins are important for several reasons. The toxins are still a problem in connection with infectious diseases and they may be used as bioterror weapons. In contrast, it seems that they could be excellent tools in medicine, either used for targeted cell killing or to introduce epitopes or proteins into cells. Many efforts were put together during the last years to develop immunotoxins effectively working in cancer therapy [277,278,279]. In this field, knowledge about AB-toxin transport from the ER to the cytosol and how they cope with ER stress and the unfolded protein response might be also very important. It has been demonstrated that ricin A chain inhibits UPR and in this way increases its own cytotoxicity [280]. Thus, it was suggested that this mode of ricin action enhances its potential as a therapeutic agent in solid tumours. These results are in agreement with previous experiments demonstrating that RTA suppressed induction of the UPR in yeast [281]. However, in contrast to these studies, it was shown that ricin, the Shiga and cholera toxins, can induce one or more of the UPR pathways in mammalian cells [282,283,284,285]. Explanation of these complicated mechanisms would highlight the role of ERAD, ER stress and UPR in responses to infections and the toxin activities.
